# Laser Lesion in the Mouse Visual Cortex Induces a Stem Cell Niche-Like Extracellular Matrix, Produced by Immature Astrocytes

**DOI:** 10.3389/fncel.2020.00102

**Published:** 2020-05-21

**Authors:** Lars Roll, Ulf T. Eysel, Andreas Faissner

**Affiliations:** ^1^Department of Cell Morphology and Molecular Neurobiology, Faculty of Biology and Biotechnology, Ruhr University Bochum, Bochum, Germany; ^2^International Graduate School of Neuroscience, Ruhr University Bochum, Bochum, Germany; ^3^Department of Neurophysiology, Faculty of Medicine, Ruhr University Bochum, Bochum, Germany

**Keywords:** astrocyte, chondroitin sulfate, cortical plasticity, extracellular matrix, neural stem cell, niche, regeneration, tenascin

## Abstract

The mammalian central nervous system (CNS) is characterized by a severely limited regeneration capacity. Comparison with lower species like amphibians, which are able to restore even complex tissues after damage, indicates the presence of an inhibitory environment that restricts the cellular response in mammals. In this context, signals provided by the extracellular matrix (ECM) are important regulators of events like cell survival, proliferation, migration, differentiation or neurite outgrowth. Therefore, knowledge of the post-lesional ECM and of cells that produce these factors might support development of new treatment strategies for patients suffering from traumatic brain injury and other types of CNS damage. In the present study, we analyzed the surround of focal infrared laser lesions of the adult mouse visual cortex. This lesion paradigm avoids direct contact with the brain, as the laser beam passes the intact bone. Cell type-specific markers revealed a distinct spatial distribution of different astroglial subtypes in the penumbra after injury. Glial fibrillary acidic protein (GFAP) as marker for reactive astrocytes was found broadly up-regulated, whereas the more immature markers vimentin and nestin were only expressed by a subset of cells. Dividing astrocytes could be identified via the proliferation marker Ki-67. Different ECM molecules, among others the neural stem cell-associated glycoprotein tenascin-C and the DSD-1 chondroitin sulfate epitope, were found on astrocytes in the penumbra. *Wisteria floribunda* agglutinin (WFA) and aggrecan as markers for perineuronal nets, a specialized ECM limiting synaptic plasticity, appeared normal in the vicinity of the necrotic lesion core. In sum, expression of progenitor markers by astrocyte subpopulations and the identification of proliferating astrocytes in combination with an ECM that contains components typically associated with neural stem/progenitor cells suggest that an immature cell fate is facilitated as response to the injury.

## Introduction

Lesions of the mammalian central nervous system (CNS) are characterized by a limited regeneration capacity. In the light of patients’ recovery it is of interest to understand the molecular and cellular mechanisms underlying this limitation on the one hand and to identify the potential of cells in this region on the other hand. In this study, laser-induced lesions of the mouse visual cortex were employed as a model for brain injury and the lesion response was assessed with a focus on the fate of astrocytes and on different factors of the extracellular matrix (ECM). The visual cortex is easily accessible for manipulation and has been used for several plasticity studies (Eysel and Schweigart, 1999; Eysel et al., 1999). Originally established in cats and rats, the visual cortex lesions were also adapted to mice, where the lesion is inflicted by an infrared laser (Roll et al., 2012). In this lesion paradigm, direct contact of the brain with surgical instruments and exposure to the environment are avoided, as the laser beam passes the intact bone. This provides well-defined conditions and a direct infection of the brain with contaminating pathogens can be excluded. In the close surround of laser-induced lesions, reduced blood flow comparable to the penumbra of ischemic infarctions has been described (Kiessling et al., 1990; Lindsberg et al., 1991). The well-defined border of the lesion core enables the post-lesional study of the vulnerable surround with high spatial precision.

Astrocytes respond to injuries and participate in a process called reactive gliosis. They produce cytokines and ECM molecules, are involved in glial scar formation and can show stem cell properties under specific circumstances (Robel et al., 2011; Pekny and Pekna, 2014). Therefore astrocytes are interesting as a potential source for new cells that might contribute to new networks.

Cell behavior and the cell fate are tightly regulated by external signals. Accordingly the ECM, a complex network that serves as reservoir for a multitude of factors, and its composition are of interest. The importance of the ECM for CNS function is undisputed, for example as one part of the neural stem cell niche (Reinhard et al., 2016). A factor that is associated with immature neural cells is the glycoprotein tenascin-C (Tnc). During development it is expressed by astrocytes, before it is downregulated and restricted to the adult neural stem cell niche (Faissner et al., 2017). Tnc regulates processes such as adhesion, migration, neurite outgrowth, but also modulates the immune system (Loers and Schachner, 2007; Piccinini and Midwood, 2012). Interestingly, Tnc is re-expressed in the diseased CNS (Roll et al., 2012; Roll and Faissner, 2019). Typically ECM molecules are glycosylated and these carbohydrate modifications can modulate the properties of the carrier molecules, for example resulting in an altered interaction of different factors (reviewed by Hayes and Melrose, 2018). Specific glycoepitopes, which can be detected by monoclonal antibodies (mAbs), have been identified that show distinct expression patterns and therefore are expected to fulfill specific functions. The DSD-1 (dermatan sulfate-dependent 1) chondroitin sulfate epitope, recognized by mAb 473HD, is associated with neural stem cells and its blockage has been shown to affect proliferation of neural stem cells *in vitro* (von Holst et al., 2006). Glycoepitopes of the LewisX (LeX; also SSEA-1) type are trisaccharides. Specific antibodies are available that recognize LeX in distinct contexts: mAb 487^*LeX*^ detects terminal LeX motifs, whereas mAb 5750^*LeX*^ binds internal motifs (Hennen et al., 2011). Here, both antibodies have been shown to label different subpopulations of neural stem/progenitor cells. A related epitope, detected by the mAb 4860, has been found on cells of the oligodendrocyte lineage in the developing CNS (Czopka et al., 2009). Perineuronal nets (PNNs) are a specialized form of matrix that enwraps subtypes of neurons and is associated with plasticity restriction in the adult CNS. In the context of potentially increased plasticity after brain injury, analysis of PNN integrity can shed light on the underlying mechanisms. Indeed, PNN degradation in the diseased CNS has been described, but seems to depend on the type of damage (reviewed by Bozzelli et al., 2018).

## Materials and Methods

### Animals

129S2/SvPasCrl (RRID:IMSR_CRL:287) mice were originally obtained from Charles River and held in the animal facility of the Ruhr University Bochum (Germany).

### Infrared Laser Lesion of the Visual Cortex

All procedures were performed in accordance with the German law (§15 TierSchG) and approved by the animal protection commission of the Landesamt für Natur, Umwelt und Verbraucherschutz Nordrhein-Westfalen (file number 84-02.04.2012.A363). Laser lesions were performed according to a standardized protocol (Roll et al., 2012). In short, young adult mice (12 weeks old) were anesthetized with 65 mg ketamine, 13 mg xylazine and 2 mg acepromazine (all CP-Pharma, Burgdorf, Germany) per kg body weight (i.p.). Body temperature was stabilized by a heating pad. The scalp covering the cortex was cut with a scalpel, and the bone was drilled thin. A row of overlapping, round lesions (each 0.5 mm in diameter, 2 W, 810 nm) was inflicted to the right visual cortex through the wet (PBS), intact bone by an infrared laser (OcuLight SLx; Iris Medical/Iridex, Mountain View, CA, United States). Eventually, an area 2 mm long (A-P) and 0.5 mm wide (M-L), located 1 mm anterior from lambda suture and 1 mm lateral from sagittal suture, was affected by necrosis (lesion core). The skin wound was closed with tissue glue and the mice were allowed to recover in their cage under close monitoring.

### Tissue Preparation and Immunohistochemistry

3, 7, 14, or 28 days post-lesion (dpl), animals were anesthetized with 65 mg ketamine, 13 mg xylazine and 2 mg acepromazine per kg body weight (i.p.). The heart was exposed and the mouse was transcardially perfused for 5 min with heparin-supplemented (Liquemin N 25000, Roche, Mannheim, Germany; diluted 1:500) physiological salt solution (0.9% NaCl) to remove blood from the vascular system. Afterward animals were perfused for 15 min with 4% PFA to fix the tissue. The brain was dissected and fixed in 4% PFA for 24 h at 4°C, before it was transferred into 30% sucrose solution for cryoprotection. Finally, the tissue was mounted in Tissue-Tek (Sakura Finetek, Torrance, CA, United States) on dry ice and stored at −70°C.

Cryosections were prepared using a cryostat. 20-μm-thick frontal sections were collected either on Superfrost Plus microscope slides (Thermo Scientific, Braunschweig, Germany) or free-floating with a brush in cold PBS, supplemented with 1 mM EDTA. Tissue on glass was stored at −70°C, free-floating tissue was transferred into cryo vials filled with 1 mL mixture of glycerol and 30% (w/v) sucrose in PBS (1:1), supplemented with 1 mM EDTA.

Two immunohistochemistry protocols, for free-floating slices or slices on glass, were applied, depending on the antibody. Usually, stainings of ECM molecules were performed with free-floating slices in 96-well-plates. The slices were moved from one well to the next with a curved glass pipette. Slices were incubated in blocking solution (PBS supplemented with 1% bovine serum albumin, 5% goat serum and 0.5% Triton X-100) for 1 h at RT, in primary antibodies over night at 4°C, washed with PBS (3 min × 15 min), treated with secondary antibodies (diluted in blocking solution w/o Triton X-100) for 3 h, followed by washing in PBS (3 min × 15 min) before mounting in Immu-Mount (Thermo Fisher Scientific, Waltham, MA, United States) at RT. Alternatively, slices on glass slides were thawed and dried for 15 min at RT and washed in PBS. For PNN stainings, slices were heated in citrate buffer and kept at 100°C for 5 min. After 10 min the buffer was cooled on ice and the slices were washed in PBS for 5 min. The tissue was surrounded with Roti-Liquid barrier marker (Carl Roth, Karlsruhe, Germany). The following steps were performed in a dark, humidified chamber: after blocking for 1 h at RT, the primary antibody was applied over night at 4°C. Unbound antibodies were removed by washing in PBS (3 min × 5 min), before secondary antibodies were applied for 1 h, followed by washing in PBS (3 min × 5 min) and mounting in Immu-Mount (all at RT). Antibodies are listed in Tables 1, 2. Cell nuclei were counterstained with Hoechst 33258 or To-Pro-3.

**TABLE 1:** Primary antibodies used for immunohistochemical analysis (IHC) and Western Blot (WB).

	Target	Species	Dilution	Reference/RRID
**Antibody**				
473HD	DSD-1 epitope	Rat IgM	IHC:	Faissner et al., 1994
			1:250	
4860	Glycolipid	Rat IgM	IHC:	Czopka et al., 2009
			1:200	
487^*LeX*^ (L5)	LeX epitope	Rat IgM	IHC:	Streit et al., 1990
			1:350	
5750^*LeX*^	LeX epitope	Rat IgM	IHC:	Hennen et al., 2011
			1:50	
Aggrecan	Aggrecan	Rabbit poly.	IHC:	Merck Millipore
			1:300	RRID:AB_90460
βIII Tubulin	βIII Tubulin	Mouse IgG	IHC:	Sigma-Aldrich
			1:250	RRID:AB_477590
DM1A	α Tubulin	Mouse IgG	WB:	Sigma-Aldrich
			1:10,000	RRID:AB_477593
GFAP	Glial fibrillary acidic protein	Mouse IgG	IHC:	Sigma-Aldrich
			1:300	RRID:AB_477010
GFAP	Glial fibrillary acidic protein	Rabbit poly.	IHC:	DAKO
			1:200	RRID:AB_10013382
GFAP	Glial fibrillary acidic protein	Rat IgG	IHC:	Merck Millipore
			1:400	RRID:AB_211868
Iba1	Ionized calcium-binding adapter molecule 1	Goat IgG	IHC:	Abcam
			1:125	RRID:AB_2224402
Ki-67	Nuclei of dividing cells	Mouse IgG	IHC:	Novocastra/Leica
			1:20	RRID:AB_563841
Ki-67	Nuclei of dividing cells	Rabbit IgG	IHC:	Thermo Scientific
			1:50	RRID:AB_2341197
Nestin	Nestin	Mouse IgG	IHC:	Merck Millipore
			1:250	RRID:AB_94911
Tnc (Kaf 14.1)	Tenascin-C	Rabbit poly.	IHC: 1:250	Faissner and Kruse, 1990
			WB: 1:300	
Tnc (Kaf 12F)	Tenascin-C	Rabbit poly.	IHC:	Faissner and Kruse, 1990
			1:250	
Vimentin	Vimentin	Mouse IgM	IHC:	Sigma-Aldrich
			1:200	RRID:AB_261856
**Lectin**				
WFA (biotinylated)	Perineuronal nets	*Wisteria floribunda*	1:100	Sigma-Aldrich
				L1516-2MG

**TABLE 2:** Secondary antibodies used for immunohistochemical analysis (IHC) and Western blot (WB).

Antibody	Species	Dilution	RRID
Anti goat, AF488	Donkey	1:300; IHC	Dianova RRID:AB_2336933
Anti mouse IgM, AF488	Goat	1:300; IHC	Dianova RRID:AB_2338849
Anti mouse IgG, Cy3	Goat	1:300; IHC	Dianova RRID:AB_2338687
Anti mouse IgG + IgM, AF488	Goat	1:300; IHC	Dianova RRID:AB_2338844
Anti mouse IgG + IgM, Cy3	Goat	1:300; IHC	Dianova RRID:AB_2338686
Anti mouse IgG + IgM, AF647	Goat	1:300; IHC	Dianova RRID:AB_2338908
Anti mouse IgG + IgM, Cy3	Rabbit	1:300; IHC	Dianova RRID:AB_2340139
Anti rabbit IgG, AF488	Goat	1:300; IHC	Dianova RRID:AB_2338049
Anti rabbit IgG, AF488	Donkey	1:300; IHC	Dianova RRID:AB_2313584
Anti rabbit IgG, Cy3	Goat	1:300; IHC	Dianova RRID:AB_2338003
Anti rabbit IgG, AF647	Goat	1:300; IHC	Dianova RRID:AB_2338078
Anti rat IgG + IgM, AF488	Goat	1:300; IHC	Dianova RRID:AB_2338357
Anti rat IgM, FITC	Goat	1:300; IHC	Dianova RRID:AB_2338198
Anti rat IgM, Cy3	Goat	1:300; IHC	Dianova RRID:AB_2338249
Anti mouse IgG + IgM, HRP	Goat	1:10,000; WB	Dianova RRID:AB_2338505
Anti rabbit IgG, HRP	Goat	1:10,000; WB	Dianova RRID:AB_2307391

### *In situ* Hybridization

*In situ* hybridization was performed according to a protocol described by N. P. Pringle and W. D. Richardson (Wolfson Institute for Biomedical Research, London, United Kingdom). The composition of buffers and solutions is listed in Supplementary Table 1. In short, 20-μm-thick slices on glass slides were thawed and dried for 10 min at RT. Digoxigenin-labeled RNA riboprobes (sequence: see Supplementary Table 2) were diluted 1:500 in hybridization mix and 250 μL of this solution were given onto each slice. The solution was covered with cover slips to avoid evaporation when the tissue was incubated for hybridization at 65°C over night in a humidified (2x SSC, 50% formamide) chamber. The cover slips were carefully removed by rinsing with wash buffer in a cuvette. Subsequently, two washing steps with pre-heated wash buffer at 65°C and three steps with MABT buffer at RT followed. The slices were transferred to a humidified (aqua dest) chamber and encircled with barrier marker. 150 μL blocking solution were added for 1 h at RT and subsequently replaced by 150 μL anti-DIG-AP (Roche Diagnostics, Mannheim, Germany) antibody solution, which was incubated at 4°C over night. After three washing steps with MABT buffer (each 10 min) at RT the samples were incubated in pre-developing buffer (2 min × 5 min at RT). Afterward, 200 μL developing solution were applied for at least 2 h at 37°C in the dark. Staining induced by the alkaline phosphatase was controlled under the microscope and stopped by incubation in aqua dest for 5 min. The slices were mounted with Immu-Mount and stored at 4°C.

### Microscopy

After *in situ* hybridization, tissue was documented with a Leica MZ6 stereomicroscope (Leica Microsystems, Wetzlar, Germany), for higher magnifications with an Axioplan 2 microscope (Carl Zeiss, Oberkochen, Germany). Immunohistochemical stainings were documented with an Axio Zoom.V16, for higher magnifications with an Axioplan 2 or an LSM 510 META (all Carl Zeiss, Oberkochen, Germany). Images were exported and processed with Adobe Photoshop, Adobe Illustrator (both CS6; Adobe, Dublin, Ireland) and ImageJ (Fiji; Schindelin et al., 2012).

### Quantitative Analysis

Cells in immunohistochemical stainings were counted manually using the cell counter tool in ImageJ (Fiji; Schindelin et al., 2012). Data were analyzed with Excel software (Office 2010; Microsoft Corporation, Redmond, WA, United States) and visualized with Adobe Illustrator (CS6; Adobe, Dublin, Ireland). To quantify the relative proliferation rate of reactive astrocytes (GFAP/Ki-67 double-positive cells in relation to all GFAP-positive cells), the cells in the penumbra of three brains (*n* = 3; three slices per brain) were counted. The total number of cells positive for GFAP, vimentin and nestin was determined by counting the cells in a defined area of 500 μm x 500 μm, directly adjacent to the border of the necrotic lesion core (*n* = 3; one slice per brain; 20-μm-thick slices).

### SDS-Polyacrylamide Gel Electrophoresis (SDS-PAGE), Western Blot

1-mm-thick frontal sections of fresh brain tissue were sectioned with razor blades in cooled PBS (4°C). Each section was divided into four parts of equal size by a horizontal and a vertical cut. These parts were individually shock-frozen in reaction tubes on dry ice and stored at −70°C. The composition of buffers and solutions used for SDS-PAGE and Western blot is listed in Supplementary Table 3. In short, proteins were isolated by addition of 1 mL lysis buffer under trituration, first with a 1,000 μL pipette and subsequently with a fine insulin syringe. Samples were incubated on ice for 1 h, centrifuged (15 min at 4°C) and the supernatant was isolated and stored at −20°C before use. For SDS-PAGE, 15 μL of the supernatant were mixed with 5 μL loading buffer, heated (95°C for 5 min) and cooled on ice. Proteins were separated according to their molecular weight in polyacrylamide gels by an electric field (20 mA) for ca. 1 h in an electrophoresis unit (Hoefer, Heidelberg, Germany). Subsequently, proteins were transferred onto a PVDF membrane (Carl Roth, Karlsruhe, Germany) in an electric field of 75 mA for 1.5 h. The membrane was blocked with 5% milk powder in TBST for 1 h at RT. Primary antibodies were diluted in 5% milk powder in TBST and incubated at 4°C over night. After three washing steps with TBST (each 5 min) the secondary antibody was incubated for 1 h. Three washing steps with TBST and one step with TBS followed, before 5 mL of the ECL solution (Carl Roth, Karlsruhe, Germany) were applied for 5 min. The membrane was documented with a MicroChemi imaging system (DNR, Neve Yamin, Israel).

### Reverse Transcription Polymerase Chain Reaction (RT-PCR)

1-mm-thick frontal sections of fresh brain tissue were sectioned with razor blades in cooled PBS (4°C). Each section was divided into four parts of equal size by a horizontal and a vertical cut. These parts were individually shock-frozen in reaction tubes on dry ice and stored at −70°C. RNA was isolated using the GenElute Total RNA purification kit (Sigma-Aldrich, St. Louis, MO, United States). 1 μg RNA was used for cDNA synthesis with the First strand cDNA synthesis kit (Thermo Scientific, Waltham, MA, United States). Details of the PCR buffers, primer sequences and the PCR program are provided in Supplementary Table 4. In short, PCR was performed with 1 μl of cDNA in a Mastercycler gradient (Eppendorf, Hamburg, Germany). The number of cycles was reduced from a standard of 25 to 20 for β *Actin* samples to avoid saturation effects. DNA amplicons were separated by agarose gel electrophoresis according to their size. Gels were documented with a digital camera under UV light (LTF, Wasserburg am Inn, Germany). Band intensity was measured densitometrically with ImageJ: after background subtraction, a box of equal size was drawn around each band and the “mean” value was determined using the “Measure” tool. Each value was normalized to the housekeeping gene β *Actin*. Data were analyzed with Excel software and visualized with Adobe Illustrator.

## Results

The cell fate as well as the expression of ECM molecules associated with immature neural cell populations and PNNs were assessed in the laser lesion model.

### Reactive Gliosis After Cortical Laser Lesion

**FIGURE 1:**
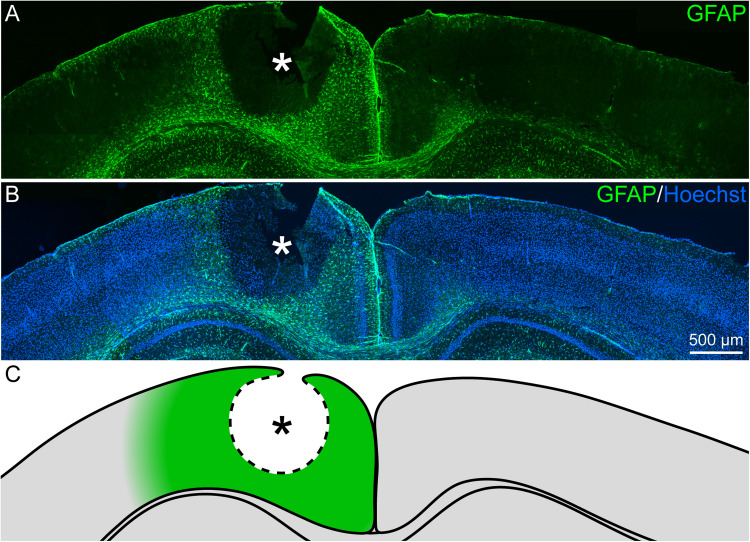
Reactive gliosis after cortical laser lesion, 3 dpl. **(A**,**B)** GFAP-positive astrocytes appeared in the penumbra of the necrotic lesion core in the adult visual cortex (frontal brain section). Under healthy conditions, GFAP was only sparsely expressed. **(C)** Scheme of the lesion that indicates the penumbra around the lesion core in green. Asterisk: lesion core; Hoechst: nuclear counterstaining; scale bar: 500 μm.

Reactive gliosis, which includes reactive astrocytes, is a hallmark in the lesion response of the CNS. Accordingly, the fate of astrocytes was examined in a first step with immunohistochemical stainings. Reactive gliosis could be observed by comparing the GFAP signal of the lesioned hemisphere with the contralateral side 3 days after laser lesion (3 dpl; Figures 1A,B). In the healthy, contralateral cortex only a faint GFAP signal in few cells was observed. The majority of these cells was found in close vicinity to the corpus callosum and in the outer cortical layers. In contrast, the penumbra was characterized by high levels of GFAP expressed by hypertrophic, reactive astrocytes that occurred in all cortical layers. This region (Figure 1C) was then analyzed in detail.

**FIGURE 2:**
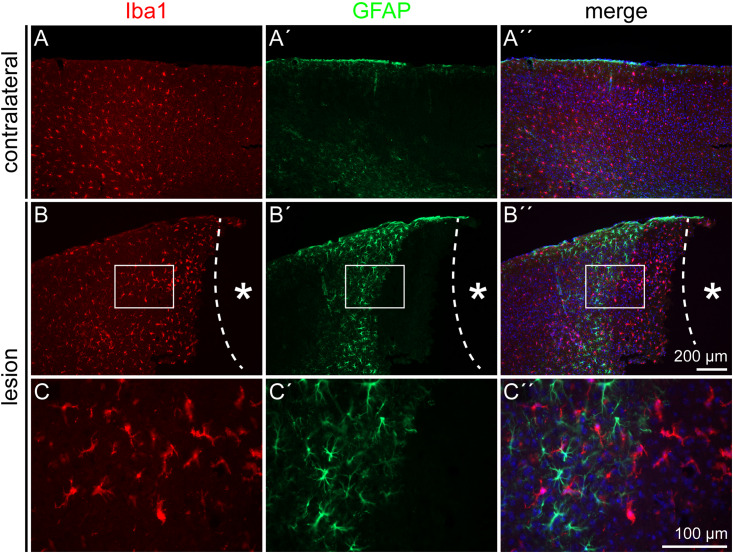
Microglia and astrocyte distribution after lesion, 3 dpl. Iba1 staining showed no colocalization with the GFAP signal. As on the contralateral side **(A–A′′)**, Iba1-positive microglia were distributed also throughout the cortex in the penumbra **(B–B′′)**. GFAP-positive astrocytes kept a distance of more than 300 μm from the lesion core, whereas Iba1-positive microglia could be found directly adjacent to it. **(C–C′′)** Show higher magnifications of the boxes in **(B–B′′)**. Asterisk: lesion core; scale bars: 200 μm **(A–B′′)**, 100 μm **(C–C′′)**.

Reactive gliosis does not only involve astrocytes, but also activated microglia cells. Iba1, a marker for resting and activated microglia, was used to detect this cell type in the penumbra. Double staining for the astrocyte marker GFAP and the microglia marker Iba1 showed distinct signals that did not overlap (Figure 2). Microglia were detected throughout the whole cortex, in the healthy as well as in the lesioned hemisphere (Figures 2A–B′′). No obvious difference in the density of Iba1 signals was observed in the lesioned hemisphere. Microglia were also present directly adjacent to the necrotic lesion core (Figures 2C–C′′). In contrast, this region was completely free of GFAP-labeled astrocytes. They appeared in high numbers in the penumbra as described above, but they were completely absent in a radius of more than 300 μm around the lesion core, with an abrupt border.

### Progenitor Marker Expression by Reactive Astrocytes

**FIGURE 3:**
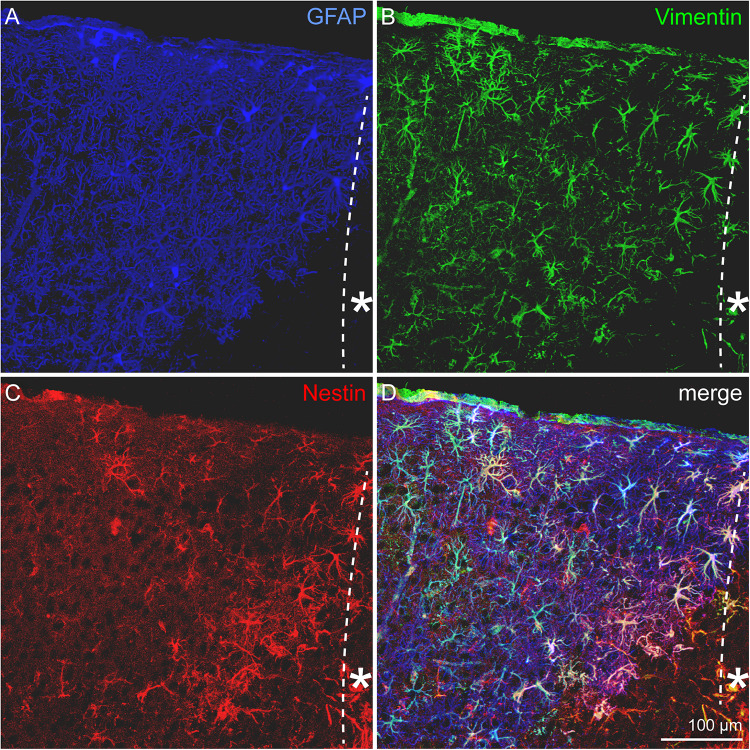
Astrocytic subpopulations in the lesioned cortex, 3 dpl. **(A)** GFAP-positive, reactive astrocytes appeared in high numbers in the penumbra. GFAP was broadly upregulated, whereas vimentin **(B)** and most strikingly nestin **(C)** were restricted to cells near the lesion core. Nearly all of the vimentin- and nestin-expressing cells were also positive for GFAP **(D)**. Asterisk: direction toward the lesion core; scale bar: 100 μm.

Astrocytes represent a heterogeneous cell type with numerous subpopulations. To assess these astroglial subtypes, distribution of the markers GFAP, nestin, and vimentin was analyzed in the penumbra. Triple staining for the markers (Figure 3) shows a prominent expression of the astrocyte marker GFAP and only a subset of the cells coexpressed vimentin. Even fewer cells were also positive for the progenitor marker nestin. We quantified the cells in a defined area of 500 μm x 500 μm adjacent to the border of the necrotic lesion core and found that 71.2 ± 5.5 cells expressed GFAP, 30.7 ± 11.0 cells expressed vimentin and only 16 ± 13 cells expressed nestin in 20 μm-thick slices (Supplementary Figure 1). The distribution of marker-expressing cells appeared not arbitrarily. Instead, vimentin and even more obviously the nestin-positive cells were restricted to the region close to the lesion core. Outside the penumbra, cells positive for these markers are absent in the healthy cortex.

### Proliferation of Reactive Astrocytes

**FIGURE 4:**
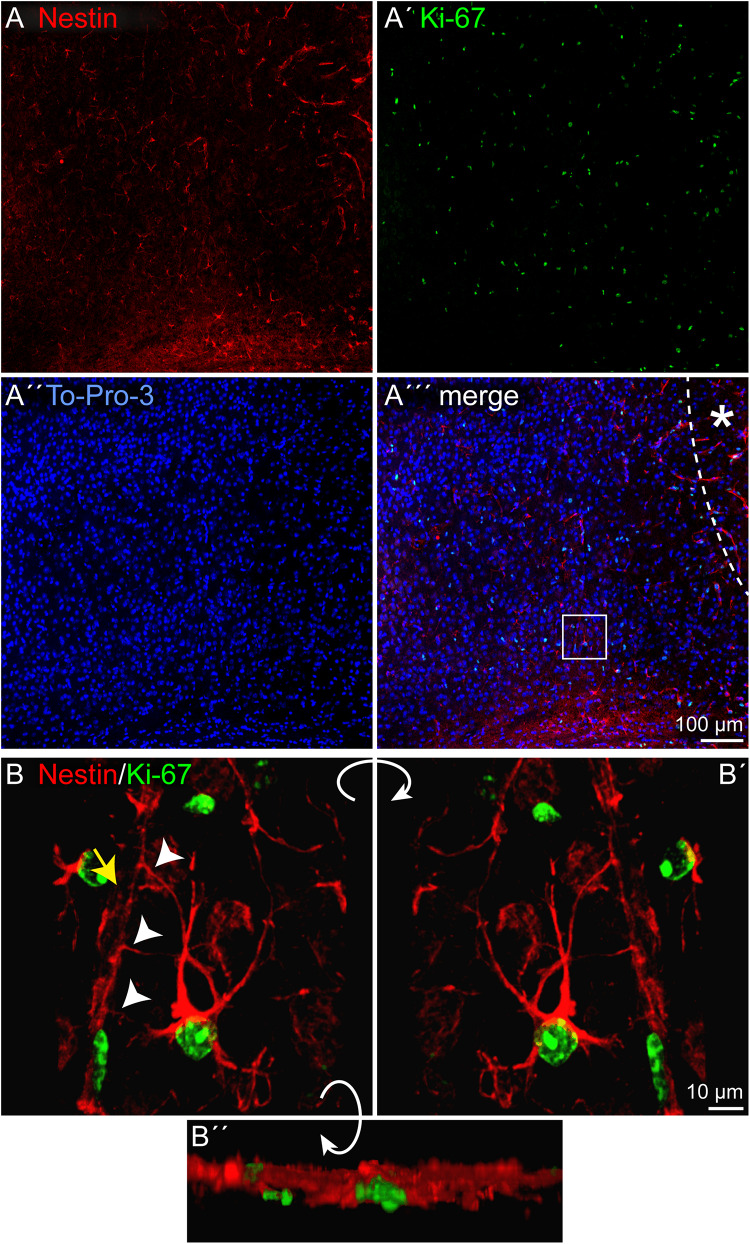
Proliferation of nestin-positive cells, 3 dpl. **(A–A′′′)** Staining of the proliferation marker Ki-67 revealed numerous proliferating cells in the penumbra after lesion. Some of these cells were also nestin-positive. **(B–B′′)** An example of such a cell boxed in **(A′′′)** is shown after three-dimensional reconstruction of confocal images. The cell formed at least three contacts (arrowheads) to vasculature (yellow arrow). Asterisk: direction toward the lesion core; scale bars: 100 μm **(A–A′′′)**, 10 μm **(B–B′′)**.

**FIGURE 5:**
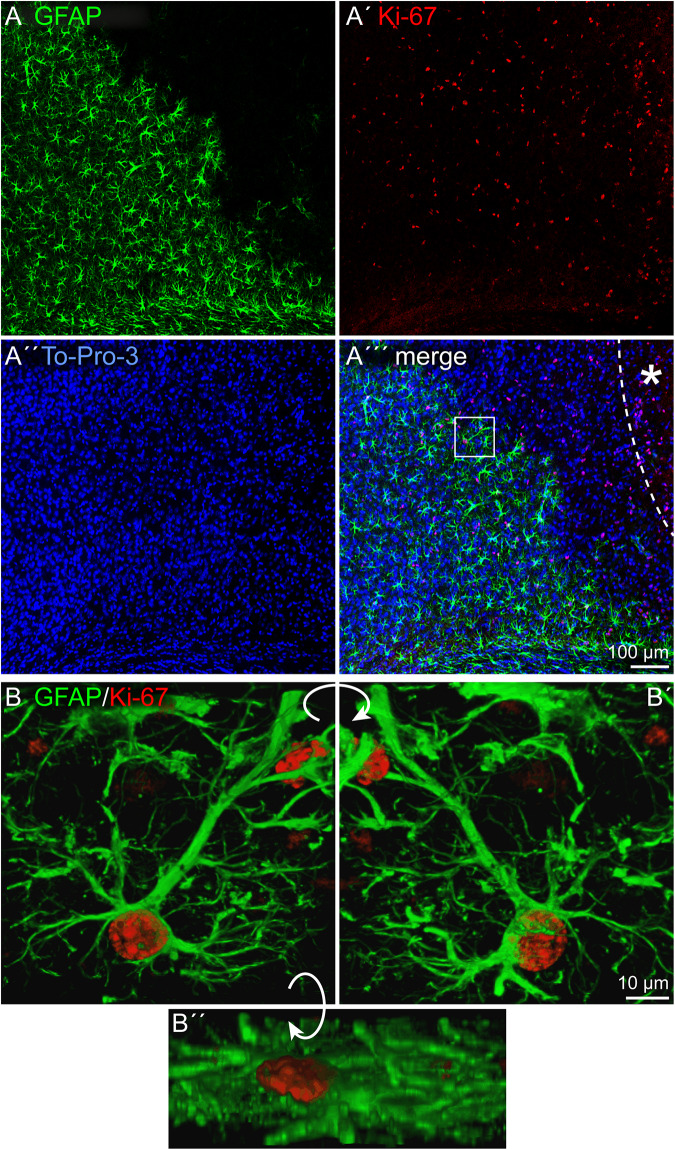
Proliferation of reactive astrocytes, 3 dpl. **(A–A′′′)** Immunohistochemical stainings for Ki-67 labeled numerous proliferating cells in the penumbra after lesion. Some of these cells were also GFAP-positive. **(B–B′′)** An example of a proliferating astrocyte (boxed in **A′′′**) is shown after three-dimensional reconstruction of confocal images. Asterisk: direction toward the lesion core; scale bars: 100 μm **(A–A′′′)**, 10 μm **(B–B′′)**.

Depending on the lesion paradigm, reactive astrocytes can re-enter the cell cycle. To examine if laser lesion of the mouse visual cortex induced astrocyte proliferation in the penumbra, double stainings of nestin and GFAP with the proliferation marker Ki-67 were performed. Ki-67 labels proliferating cells in all phases of the cell cycle. A huge number of cells in the lesioned cortex were positive for Ki-67 (Figures 4A–A′′′). For an unambiguous allocation of a nucleus with a certain cell to avoid false-positive results from nuclei of other cells, and therefore of a potentially different cell type, three-dimensional reconstructions of confocal images were analyzed. The fact that nestin and GFAP both are constituents of intermediate filaments and therefore part of the cytoskeleton allowed a clear allocation. Double staining of nestin and Ki-67 3 days after lesion indeed revealed proliferating cells that expressed the progenitor marker nestin. Detailed analysis of one cell shows the labeled cytoskeleton encasing the nucleus (Figures 4B–B″). The cell shown in this example had three long, fine processes that contacted a blood vessel (arrows). Also proliferating GFAP-expressing cells were identified in the penumbra, as shown by the clear coexpression of GFAP in the cytoskeleton around a Ki-67-positive nucleus (Figures 5A–B″). Quantification revealed that 1.5 ± 0.4% of the GFAP-positive, reactive astrocytes in the cortical penumbra expressed Ki-67 (Supplementary Figure 2).

### Extracellular Matrix After Lesion

**FIGURE 6:**
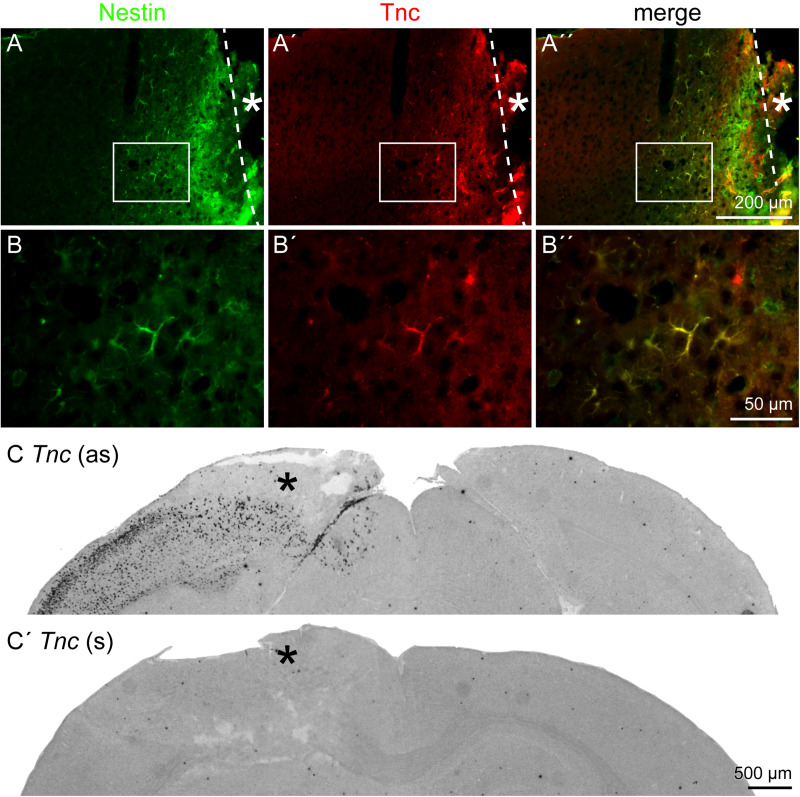
Tnc expression by reactive astrocytes, 3 dpl. **(A–B′′)** Tnc was expressed by nestin-positive reactive astrocytes in the penumbra. **(B–B′′)** Higher magnifications of the boxed region in **A–A′′**. **(C,C′)** *In situ* hybridization revealed a prominent re-expression of *Tnc* mRNA in the lesioned cortex, whereas no expression was found on the contralateral side. The sense riboprobe (s), used as a negative control, showed no unspecific staining. Asterisk: lesion core; scale bars: 200 μm **(A–A′′)**, 50 μm **(B–B′′)**, 500 μm **(C,C′)**.

After characterization of the cell fate in the penumbra, with a focus on the astrocytic lineage, the extracellular environment was analyzed. Tnc was re-expressed in the penumbra by nestin-positive, reactive astrocytes (Figures 6A–B′′). Immunohistochemical analyses in the lesioned brain are always critical with regard to unspecific staining and strong background due to the damaged tissue containing cell debris, immune cells and other factors. *In situ* hybridization independently confirmed a prominent re-expression of *Tnc* mRNA in the penumbra after 3 days, whereas no expression was detected on the contralateral side (Figure 6C). The negative control with the sense riboprobe showed no staining in the whole brain slice (Figure 6C′). We performed RT-PCR and Western blot as pilot experiments with a time series to assess Tnc expression independently (*n* = 1; Supplementary Figure 3). RT-PCR revealed an increase in relative *Tnc* expression in the lesioned hemisphere 3 dpl (fivefold increase compared to the healthy control and to the contralateral side). Expression levels on mRNA level returned to normal values already 7 dpl. In contrast, the Western blot showed stronger signals at 3 dpl and also at 7 dpl, indicating that the increase in Tnc protein levels exceeded the short upregulation seen on mRNA level (Supplementary Figure 3).

**FIGURE 7:**
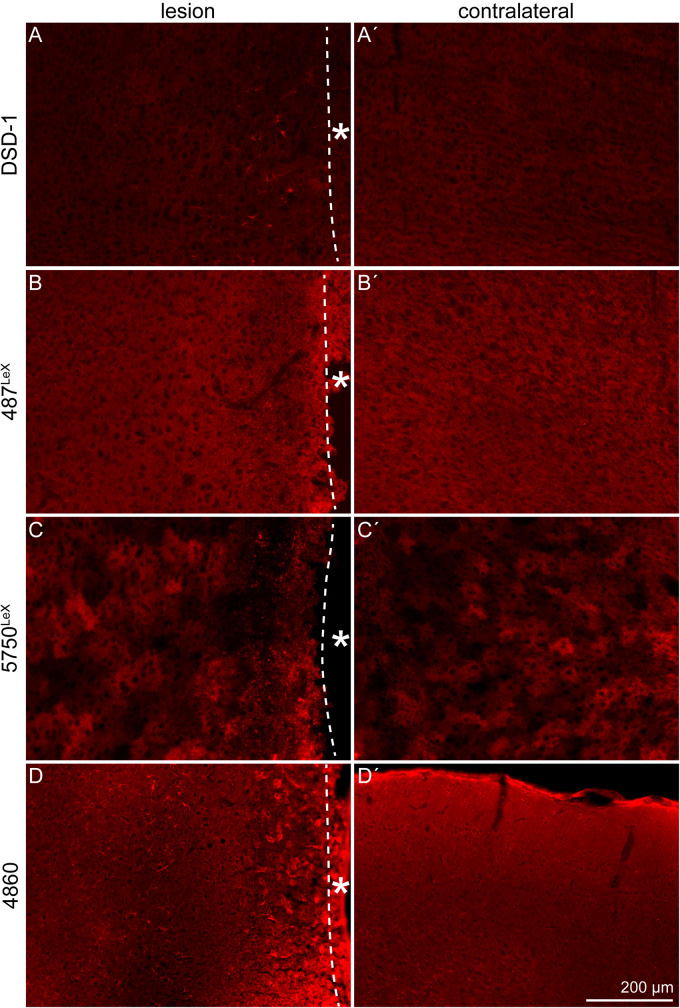
Expression of the DSD-1, 487^*LeX*^, 5750^*LeX*^, and 4860 glycoepitopes, 3 dpl. **(A,A′)** The DSD-1 glycoepitope was detected on cells, restricted to the penumbra. **(B,B′)** Staining with mAb 487^*LeX*^ did not show a lesion-specific expression of the epitope. **(C,C′)** The 5750^*LeX*^ staining revealed a characteristic, patchy expression pattern that was not altered in the penumbra. **(D,D′)** The 4860 epitope was found on distinct cells in the penumbra, in contrast to a diffuse staining in the healthy hemisphere. Asterisk: direction toward the lesion core; scale bar: 200 μm.

The expression of the stem cell-related DSD-1 glycoepitope, detected by the mAb 473HD, and other glycoepitopes in the penumbra was examined by immunohistochemical analyses. The DSD-1 was detected in the penumbra, on cells near the lesion core (Figures 7A,A′). The expression pattern of LeX-type glycoepitopes and a related epitope recognized by mAb 4860 was analyzed in a next step. mAb 487^*LeX*^ led to a diffuse signal and did not reveal a lesion-specific expression of its epitope (Figures 7B,B′). 5750^*LeX*^ staining showed a characteristic, patchy expression pattern. This was also not altered in the penumbra, except at the direct border to the lesion core (Figures 7C,C′). In addition to a diffuse staining in both hemispheres, the 4860 epitope was found on distinct cells in the penumbra that showed an astrocytic morphology (Figures 7D,D′), similar to the cells stained with GFAP earlier.

**FIGURE 8:**
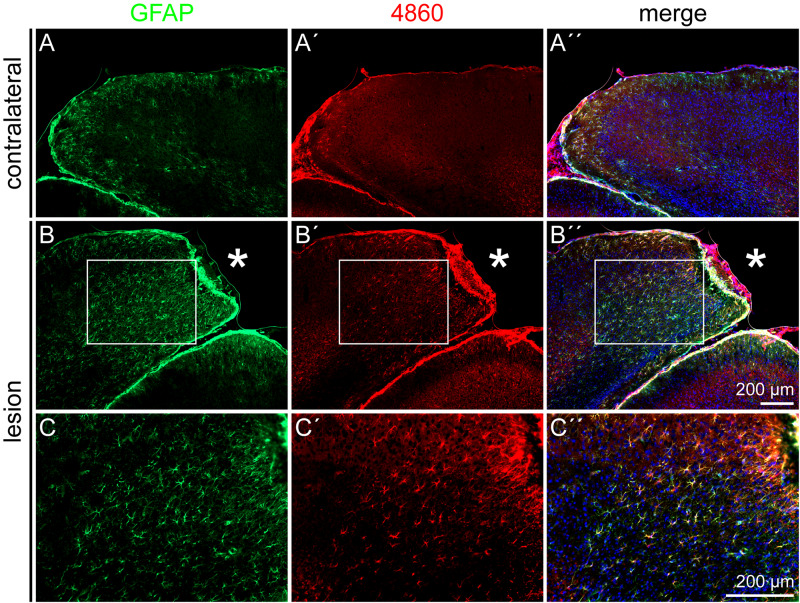
4860 glycoepitope expression on astrocytes after lesion, 3 dpl. **(A–A′′)** In the contralateral hemisphere, mAb 4860 labeled only few GFAP-positive cells. **(B–C′′)** In contrast, the penumbra was rich in 4860-positive cells, representing a subpopulation of GFAP-positive astrocytes. **(C–C′′)** Higher magnifications of the boxed region in **B–B′′**. Asterisk: lesion core; scale bars: 200 μm.

The DSD-1 epitope is associated with GFAP- and nestin-positive cells after laser lesion (Roll et al., 2012). To confirm also the astroglial identity of 4860 epitope-expressing cells, double stainings of GFAP and the 4860 epitope were performed (Figure 8). In the healthy cortex on the contralateral side, mAb 4860 only stained very few cells. These were double-positive for GFAP (Figures 8A–A′′). In contrast, the penumbra contained a large number of 4860-positive cells, distributed throughout all cortical layers. These cells represented a subpopulation of GFAP-positive astrocytes (Figures 8B–B′′). As shown in a higher magnification, not all GFAP-positive cells coexpressed the 4860 epitope, instead the epitope expression was restricted to astrocytic subpopulations (Figures 8C–C′′).

### Perineuronal Nets After Lesion

**FIGURE 9:**
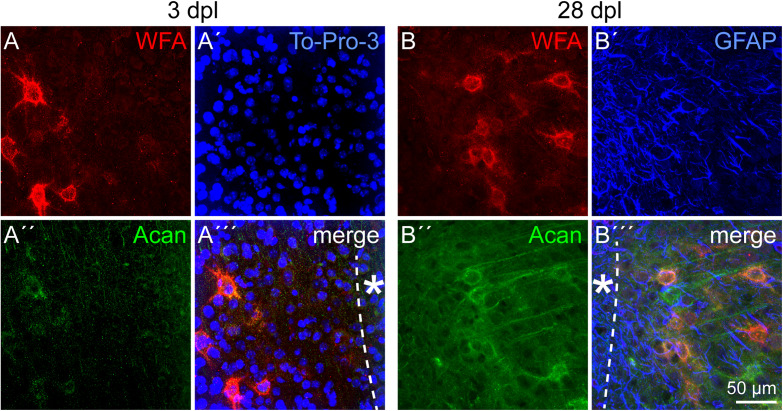
PNNs in the lesioned visual cortex. **(A–A′′′)** WFA intensely stained PNNs in the penumbra 3 dpl. Aggrecan (Acan) signals appeared more diffuse. **(B–B′′′)** The same was observed 28 dpl, when morphologically intact PNNs could still be detected. GFAP-positive astrocytes **(B′–B′′′)** indicate the high degree of reactive gliosis in the region where the PNN markers were still detectable. Asterisk: direction toward the lesion core; scale bar: 50 μm.

Beside ECM related to immature neural cell types, PNNs as a specialized form of ECM were immunohistochemically analyzed. Detected by double stainings of the two PNN markers *Wisteria floribunda* agglutinin (WFA) and aggrecan (Acan), PNNs appeared with a punctate staining pattern around individual neurons in the cortex (Figure 9). As the relative intensity of individual PNN markers can differ, also depending on the brain region, both markers were used. In contrast to WFA staining, which produced a clear staining pattern with intensely stained PNNs, the Acan staining appeared less prominent, at least in the cortex. In the penumbra of the laser lesion, PNN markers were not affected 3 dpl (Figures 9A–A′′′). The same was true 28 dpl, when morphologically intact PNNs were labeled (Figures 9B–B′′′). The location of GFAP-positive astrocytes shows the high degree of reactive gliosis. The presence of PNN markers in this environment suggests that PNNs were relatively stable in the penumbra.

## Discussion

### Heterogeneous Subpopulations of Astrocytes After Lesion

Reactive astrocytes play a crucial role in the lesion response of the CNS. They are involved in the formation of the glial scar and secrete a variety of ECM and other signaling molecules like cytokines (Dowell et al., 2009). In addition, reactive astrocytes can show characteristics of neural stem/progenitor cells. To assess the cell fate of reactive astrocytes after laser lesion, the expression of GFAP, vimentin, and nestin was immunohistochemically analyzed by triple stainings (Figure 3). In the healthy CNS, GFAP is expressed by astrocyte subpopulations (Sofroniew and Vinters, 2010), vimentin is present in the radial glia type of neural stem cells during development (Bignami et al., 1982) and nestin is the prototypical neural stem/progenitor cell marker (Dahlstrand et al., 1995). Under pathological conditions, all three markers can be expressed by reactive astrocytes (Robel et al., 2011). The observed expression pattern in our laser lesion model with broad GFAP expression, fewer cells coexpressing vimentin and only very few cells adjacent to the lesion core that were also positive for nestin suggests the emergence of distinct astrocytic subpopulations. Similar results have been obtained in the rat model after laser lesion of the visual cortex (Sirko et al., 2009). Regional progenitor marker expression that is limited to the close vicinity of the lesion site has also been detected in other lesion models, for example after spinal cord injury (SCI) and cryogenic traumatic brain injury (White et al., 2010; Kim et al., 2012; Wanner et al., 2013). Astroglial expression of progenitor markers can reflect an immature state of the cells. If they indeed re-enter the cell cycle depends on the type of damage. For example, in many models of long-term neurodegenerative diseases such as Alzheimer’s, proliferation is not or only rarely observed (Ben Haim et al., 2015). In contrast, proliferating astrocytes have been described in many cases after acute damage with blood–brain barrier disruption (Dimou and Götz, 2014).

In our laser lesion model, proliferating nestin- as well as GFAP-positive cells were detected by double stainings with the proliferation marker Ki-67 (Figures 4, 5). The fact that astrocytes proliferate does not mean that they form new neurons and replace lost cells after injury. Under many conditions, neurogenesis from reactive astrocytes has not been observed *in vivo* (Götz et al., 2015). Progenitor marker expression seems to indicate the intrinsic potential of the cells rather than the cell fates they actually adopt in the lesioned environment. The hypothesis that the cell fate is not only a question of cell-intrinsic properties but also depends on appropriate external signals is supported by severe differences reported in the astrocytes’ potential *in vivo* and *in vitro*. Astrocytes isolated after stab wound formed neurospheres and gave rise to neurons, astrocytes, and oligodendrocytes *in vitro* but not *in vivo*, indicating an anti-neurogenic environment in the adult brain, also after damage (Buffo et al., 2008).

An important question concerns the origin of the reactive astrocytes present in the penumbra. Two mechanisms are conceivable. Activation of local astrocytes on the one hand, and attraction of astrocytes with stem/progenitor characteristics from the adult stem cell niches, namely the subventricular zone (SVZ) or subgranular zone (SGZ), on the other hand. Both mechanisms have been described for different types of CNS damage. Local activation has been detected following stab wound injury (Buffo et al., 2008). After stroke, an increased proliferation in the SVZ and an attraction of SVZ cells to the damaged area were shown (Arvidsson et al., 2002). In the present study, the organized spatial distribution of astroglial subtypes, depending on their position in the penumbra, as soon as 3 days after injury suggests that local astrocytes are activated. Otherwise, nestin-positive cells could also be expected in regions outside the penumbra, on their way from the niche to their target region. This does not exclude the possibility of additional stem/progenitor cells that might be attracted from the adult stem cell niches, but favors a model where local activation is the predominant mechanism after laser lesion.

### ECM Remodeling After Lesion

As the ECM provides important signals for the cells in the penumbra (reviewed by Roll and Faissner, 2014), its composition after laser lesion was assessed with regard to Tnc (Figure 6) and the DSD-1 glycoepitope, recognized by the mAb 473HD (Figure 7). These molecules have in common that they are expressed during CNS development and are downregulated under healthy conditions in the adult.

Tnc and the DSD-1 epitope were detected exclusively in the penumbra, and they are associated with reactive astrocytes in close vicinity of the lesion (Roll et al., 2012). *Tnc* mRNA detected by *in situ* hybridization was very prominent in the lesioned hemisphere. The area was broader than Tnc detection by immunohistochemical analysis, indicating a regulation on the translational or post-translational level. An isoform-specific Tnc expression after brain injury in the rat has been reported, with isoforms containing the FNIII domains B and D specifically upregulated after cortical injury (Dobbertin et al., 2010). The findings are also in line with other reports. Tnc is expressed in the injured human cortex and is strongly restricted to those GFAP-positive cells that were very close to the lesion site in cortical lesions of the rat (McKeon et al., 1991; Brodkey et al., 1995). Tnc and Phosphacan/RPTPβ/ζ, which can carry the DSD-1 epitope, colocalize with GFAP after SCI and entorhinal cortex lesion (Deller et al., 1997; Tang et al., 2003). Expression of the DSD-1 epitope by reactive astrocytes has been described for laser lesions of the rat visual cortex, in this case the carrier molecule RPTPβ/ζ was also found upregulated (Sirko et al., 2009). Similar results have been obtained in the injured cortex, where the short isoform of RPTPβ/ζ was upregulated most prominently (Dobbertin et al., 2003).

In addition to the DSD-1 epitope, expression of the glycoepitopes recognized by the mAbs 4860, 487^*LeX*^, and 5750^*LeX*^ was analyzed immunohistochemically (Figure 7). The 4860 epitope was found on astroglial cells, shown by double staining with GFAP (Figure 8). In the healthy CNS, the epitope is part of glycolipids on cells of the oligodendrocyte lineage that have nearly completed differentiation and have already downregulated NG2 and other earlier markers (Czopka et al., 2009). During postnatal development, the epitope is strongly expressed and shows a diffuse staining pattern in the adult cortex. Under normal conditions a colocalization of the 4860 epitope with nestin and GFAP has not been described. This leads to the question how the ectopic expression of this epitope on reactive astrocytes can be interpreted. It is obvious that under pathological conditions markers can be coexpressed that do not overlap in the healthy brain. For example, nestin and GFAP are normally not coexpressed in mature astrocytes (Götz et al., 2015). This might indicate that de- or *trans-*differentiation of reactive astrocytes proceeds slowly or is not completed compared to immature cells in the developing organism. In this context, new markers are of interest to refine the characterization by the combination of markers.

Two mAbs directed against the LeX motif in specific molecular contexts, 487^*LeX*^ and 5750^*LeX*^, were employed to investigate a potential role of the LeX structures in response to laser lesion (Figure 7). These LeX antibodies had already been used to investigate glycoepitope expression in human induced pluripotent stem cell (hiPSC)-derived human neural stem/progenitor cells, where they revealed an increased epitope expression in the later neuroepithelium and radial glia state (Kandasamy et al., 2017). In the adult brain, the LeX motif is found on SVZ stem cells (Capela and Temple, 2002). The staining patterns were not modified in the penumbra. Accordingly, neither mAb 487^*LeX*^ nor mAb 5750^*LeX*^ are promising candidates for the characterization of reactive astrocytes or other cell types in the laser lesion model. The observation that LeX epitopes are not upregulated following damage has also been described for SCI (Wanner et al., 2013). It is not trivial to assign secreted molecules of the interstitial ECM to distinct cells. Once released, the molecules can diffuse or can be enriched on the surface of the producing cell but also on other cells by expression of appropriate receptors. Expression analysis on mRNA level via *in situ* hybridization is possible, but faces the problem of potential differences between mRNA level and the actual amount of protein as a result of translational regulation or proteolytic degradation.

### Perineuronal Nets and Plasticity After Lesion

PNNs as a specialized form of ECM were investigated in the light of synaptic plasticity that might be increased after lesion to allow the formation of new networks. PNN distribution was not changed in the penumbra after cortical laser lesion (Figure 9). The PNN markers WFA and aggrecan did not show differences, neither compared to the contralateral side nor to a healthy control without lesion (data not shown). PNNs were still present 28 days after injury in a region with a dense network of GFAP-positive astrocytes, indicating massive gliosis. In contrast to stable PNN marker detection in our lesion model, a very rapid PNN degradation has been described within hours after photothrombosis (Karetko-Sysa et al., 2011), which shows the huge differences between lesion paradigms. TGF-β signaling has been identified as a critical factor for PNN degradation in different lesion models (Kim et al., 2017).

Beside plastic changes based on potential PNN destabilization, plasticity has been studied on different levels in the rat laser lesion model. In the penumbra of the lesion core an increased LTP (Mittmann and Eysel, 2001; Dohle et al., 2009), associated with a special contribution of NR2B subunits, was found in *ex vivo* – *in vitro* experiments (Huemmeke et al., 2004). Increased intracellular Ca^2+^ levels and Ca^2+^ transients – both NMDA receptor- and AMPA receptor-dependent – were detected as a molecular basis of the augmented LTP (Barmashenko et al., 2001, 2003). Furthermore, cells in the surround of the lesions showed increased excitability and reduced GABAergic inhibition (Mittmann et al., 1994). All the latter changes reinstall mechanisms that support plasticity in early postnatal development but are downregulated in adulthood. Interestingly, this corresponds to the immature marker expression profile of astrocyte subpopulations and to the ECM composition found in the penumbra. It has to be considered that PNNs enwrap only some neuronal subtypes. Predominantly, but not exclusively, parvalbumin-positive GABAergic interneurons are surrounded by PNNs (Lensjo et al., 2017). So neuronal subtypes can be specifically affected after damage and show distinct responses, which is seen by a general tendency to an increased excitation:inhibition ratio, resulting in hyperexcitability after damage (Carron et al., 2016). This observation has been confirmed for the laser lesion model in the rat by the studies mentioned above.

## Conclusion

**FIGURE 10:**
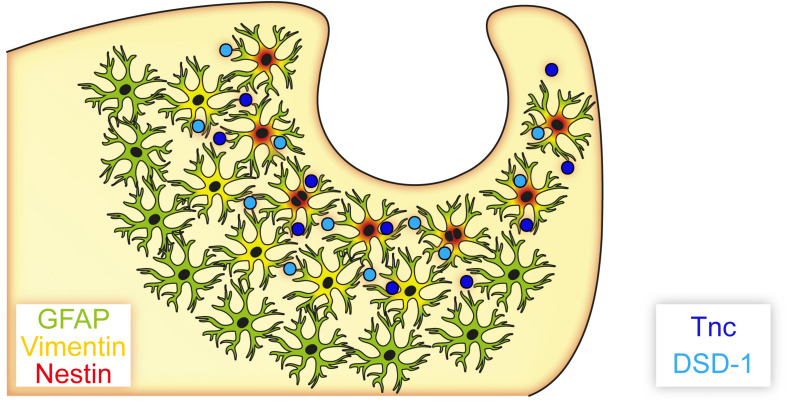
Astrocyte subpopulations and extracellular matrix after cortical laser lesion. Reactive astrocytes with distinct marker profiles were observed in a region-specific manner. GFAP-positive cells were found in a broad region of the penumbra, whereas only subpopulations of these cells also coexpressed vimentin or nestin. These markers combined with an extracellular matrix containing neural stem/progenitor cell-associated factors like Tnc and the DSD-1 epitope indicate an immature cell fate of these astrocyte subpopulations, potentially facilitated by an environment resembling parts of the neural stem cell niche.

Reactive astrocytes with potential progenitor characteristics were found in the laser lesion model (Figure 10). Cellular subpopulations coexpressing distinct progenitor markers like vimentin and nestin showed a spatial distribution in the penumbra of the necrotic lesion core, which suggests activation of local astrocytes as response to the injury. Their potential *in vivo* and *in vitro* remains to be elucidated and is a potential target for future therapeutic approaches. Manipulation of the extracellular environment could be one way to alter cell behavior. After laser lesion, the ECM composition resembled, at least in part, the ECM present in the developing organism. Presence of molecules like Tnc in the penumbra is meaningful in two respects. First, these factors might be used as markers to label astrocytic subpopulations with potential progenitor characteristics, as they are associated with the abovementioned markers GFAP, vimentin and nestin. Second, it is tempting to speculate that an ECM enriched in these molecules promotes an immature cell fate by forming a niche-like environment. Astroglial expression of the glycoepitope recognized by mAb 4860, associated with oligodendrocytes in the healthy CNS, might indicate an intrinsic potential of the reactive cells to generate new oligodendrocytes. Manipulation of these cells, via their environment or by other means, could release brain-intrinsic stem cells to improve regeneration. The fact that PNN markers were found even after weeks in the direct vicinity of the lesion suggests that synaptic plasticity might not be increased by degradation of the PNN matrix following cortical laser lesion.

A limitation of the study is the determination of the cells that produce ECM molecules and the quantification of such factors based on immunohistochemistry in the lesion environment. On the one hand, cells can bind soluble factors by expression of receptors on the cell surface, on the other hand tissue damage with cell debris, immune cell infiltration and protease activity can affect the quality of the staining. The long-term fate of reactive astrocytes including possible neurogenesis is an interesting question that remains open for future studies. Analysis of PNNs in the penumbra could be refined with regard to the ultrastructure level, as PNN function might be impaired by alterations more subtle than complete loss of typical markers.

## Data Availability Statement

All datasets generated for this study are included in the article/Supplementary Material.

## Ethics Statement

The animal study was reviewed and approved by the Animal protection commission of the Landesamt für Natur, Umwelt und Verbraucherschutz Nordrhein-Westfalen (LANUV).

## Author Contributions

LR and UE performed the experiments. AF, LR, and UE wrote and revised the manuscript.

## Conflict of Interest

The authors declare that the research was conducted in the absence of any commercial or financial relationships that could be construed as a potential conflict of interest.
