# Supplementary material for: Laser Lesion in the Mouse Visual Cortex Induces a Stem Cell Niche-Like Extracellular Matrix, Produced by Immature Astrocytes
Source: Front Cell Neurosci. 2020 May 21;14:102. doi: 10.3389/fncel.2020.00102 (PMC7253582; doi:10.3389/fncel.2020.00102)
Supplement: Supplementary file 1 [file Table_1.DOCX]

Supplementary Material

# Supplementary Table 1. Composition of buffers and solutions for *in situ* hybridization.

| **Buffer/Solution** | **Composition** |
| --- | --- |
| “Salts” (10x) | 2 M NaCl  50 mM EDTA  100 mM Tris-HCl pH 7.5  50 mM NaH_2_PO_4_ · 2 H_2_0  50 mM Na_2_HPO_4_ |
| Antibody solution | anti-Digoxigenin-AP antibody  1:1,500 in blocking solution |
| Blocking solution | MABT buffer with  2 % (w/v) blocking reagent  10 % (v/v) sheep serum |
| DEPC-treated water | add 1 mL DEPC to 1 L aqua dest,  mix and store in the dark over night,  autoclave |
| Developing solution | Pre-developing buffer with  5 % polyvinyl alcohol  0.12 mM NBT  0.11 mM BCIP  adjust pH to 9.8 |
| Hybridization mix | 10 % (v/v) “Salts” (10x)  50 % (v/v) formamide  10 % (w/v) dextran sulfate  2 % (v/v) Denhardt’s (50x)  100 µg/ml tRNA |
| MABT buffer | 100 mM maleic acid  150 mM NaCl  0.1 % (v/v) Tween 20  adjust pH to 7.5 |
| Pre-developing buffer | 100 mM Tris  100 mM NaCl  50 mM MgCl_2_  adjust pH to 9.8 |
| SSC (20x) | 3 M NaCl  0.3 M Na citrate dihydrate  adjust pH to 7.0 |
| Wash buffer | 1 % (v/v) SSC  50 % (v/v) formamide  0.1 % (v/v) Tween 20 |
