# Supplementary material for: Laser Lesion in the Mouse Visual Cortex Induces a Stem Cell Niche-Like Extracellular Matrix, Produced by Immature Astrocytes
Source: Front Cell Neurosci. 2020 May 21;14:102. doi: 10.3389/fncel.2020.00102 (PMC7253582; doi:10.3389/fncel.2020.00102)
Supplement: Supplementary file 2 [file Table_2.DOCX]

Supplementary Material

# Supplementary Table 2. mRNA sequences of the *Tnc* riboprobes (1,211 bases).

| **antisense *Tnc* riboprobe** |
| --- |
| GTCACCTGCTGTTCCACTGTATCCTTCTACCTTCAGCTTGTAGCGACTCTTGGCATCTCCAACACTGAATCTGTCGTACACAGCATAGGCAGACTCCCCATGGTCTTGTAGGTCCACCCGGAGCTCATACTGCCCTTGGGCTGTGATTTTGCTCAGGTTATCCAGTCCAAGCCAAAATTCTTCTCTGCGGTCTCCAAACCCAGCAGCATAGGCCTTCCAGTTGCGATAGAAGTCCTCACGTCCATTTTTGCGTCTCAGGAAAACAATCCATCCACCTCCATCAGAGGTCATATCACAGTAGACTTCCAGTGCTTGAGTCTTGTCACCATTTATATAGATGGTGTAGAGGCCAGAGGTAGTATCACCATTCAACATTGCTTGAGAGCAGTCCCTGGGGAATGGGTACAGGAGTCCAATTGTTGTGAAGATGGTTTGGATCAGCTTGCTCCTCAGGGACCCACTCAATGCCTGGATCCTTGCTGAGTAGTGGGTGGATGGGCTCAGGTCTGCCAGGCTGTAGGAGGTGGTGTCAGGCCCCACAATGACTTCCTTGACTGTACCATCCACAGATTCATAGACCAGGAGGTATCCAGTGACCGATGCTCGGGGAGGTCTCCAGGTAAGGAGGGCAGTTTCTGACTGAACCTCTGTAGCTGTAAATTCTCTTGGGGAATCGAGGTCTGTGGTAAACTTGGTGGCGATGGTAGAGCTCTTCTGCTGTCCTTTCTCTGCAAAGATACTCAGGATGTACTCTGTGGCAGGCTCCAGGTCATGCAGCTCGTACTCCACTGTATTTCCAGACACTGTGCGTGTAACTTCTGGCACTCTCTCCCCTGTGTAGGAGATGACATAACTGTCCACAGTGGCAATGGCTGGCTGCCACATGGCCAAGGCTTCTGAGTCTGTGATGTTGGCTATCAGAAGACCAGATGGACCATCCAGAGCTGTGATTAGAGTCCCCGAGACTGGATCGCTTTCTTCGAATCCCTTCATGGCAATCACACTGACGCGGTACTCTACACCCGGGGTAAGCTTCACCAGCCTGGTCTCAGTATCTGTTCCATCCACAGTCACCATGGACGGGGCACCTCCTGTCATAGGTACATAAGTGATCCGGAAACTCTCCACCTGAGCAGTAGGTGCCCTCCAGCTGACTGTGGCTGCATTTTCAGTGATGTCTGAGAACATGATTTCCTTCGGAGAACCCATGG |
| **sense *Tnc* riboprobe** |
| ccatgggttctccgaaggaaatcatgttctcagacatcactgaaaatgcagccacagtcagctggagggcacctactgctcaggtggagagtttccggatcacttatgtacctatgacaggaggtgccccgtccatggtgactgtggatggaacagatactgagaccaggctggtgaagcttaccccgggtgtagagtaccgcgtcagtgtgattgccatgaagggattcgaagaaagcgatccagtctcggggactctaatcacagctctggatggtccatctggtcttctgatagccaacatcacagactcagaagccttggccatgtggcagccagccattgccactgtggacagttatgtcatctcctacacaggggagagagtgccagaagttacacgcacagtgtctggaaatacagtggagtacgagctgcatgacctggagcctgccacagagtacatcctgagtatctttgcagagaaaggacagcagaagagctctaccatcgccaccaagtttaccacagacctcgattccccaagagaatttacagctacagaggttcagtcagaaactgccctccttacctggagacctccccgagcatcggtcactggatacctcctggtctatgaatctgtggatggtacagtcaaggaagtcattgtggggcctgacaccacctcctacagcctggcagacctgagcccatccacccactactcagcaaggatccaggcattgagtgggtccctgaggagcaagctgatccaaaccatcttcacaacaattggactcctgtacccattccccagggactgctctcaagcaatgttgaatggtgatactacctctggcctctacaccatctatataaatggtgacaagactcaagcactggaagtctactgtgatatgacctctgatggaggtggatggattgttttcctgagacgcaaaaatggacgtgaggacttctatcgcaactggaaggcctatgctgctgggtttggagaccgcagagaagaattttggcttggactggataacctgagcaaaatcacagcccaagggcagtatgagctccgggtggacctacaagaccatggggagtctgcctatgctgtgtacgacagattcagtgttggagatgccaagagtcgctacaagctgaaggtagaaggatacagtggaacagcaggtgac |
