# Supplementary material for: Laser Lesion in the Mouse Visual Cortex Induces a Stem Cell Niche-Like Extracellular Matrix, Produced by Immature Astrocytes
Source: Front Cell Neurosci. 2020 May 21;14:102. doi: 10.3389/fncel.2020.00102 (PMC7253582; doi:10.3389/fncel.2020.00102)
Supplement: Supplementary file 3 [file Table_3.DOCX]

Supplementary Material

# Supplementary Table 3. Buffers and solutions for SDS-PAGE and Western blot.

| **Buffer/Solution** | **Composition** |
| --- | --- |
| Blocking buffer | milk powder (5 % w/v) in TBST |
| ECL Solution | mix both solutions right before use (1:1) |
| Loading buffer (4x) | 25 ml 1 M Tris-HCl, pH 6,8  9.2 g SDS  10 ml bromophenol blue  40 ml glycerol  20 ml β-mercaptoethanol  add aqua dest to 10 mL |
| Lysis buffer | 50 mM Tris  50 mM Na acetate  60 mM n-octyl β-D-glucopyranoside  adjust pH to 8.5  add protease inhibitor mix (1:50) and PMSF (1 mM) directly before use |
| Polyacrylamide gels | stacking gel:  1.44 mL aqua dest  0.24 mL 1 M Tris-HCl (pH 6.8)  19 µL of 10 % SDS solution  0.18 mL of 40 % acrylamide  10 µL of 10 % APS  4 µL TEMED  running gel (example for 8 % gel):  2.1 mL aqua dest  1.9 mL 1 M Tris-HCl (pH 8.8)  50 µL of 10 % SDS solution  1 mL of 40 % acrylamide  34 µL of 10 % APS  9 µL TEMED |
| Running buffer (10x) | 30.2 g Tris  144.1 g glycine  10.0 g SDS  add aqua dest to 1 L |
| TBS (10x) | 250 mM Tris base  1.5 M NaCl  adjust pH to 7.4 |
| TBST | 100 mL 10x TBS  0.05 % Tween 20  add aqua dest to 1 L; pH 7.4 |
| Transfer buffer | 25 mM Tris  192 mM glycine  1 % (w/v) SDS  20 % (v/v) methanol |
