# Supplementary material for: Laser Lesion in the Mouse Visual Cortex Induces a Stem Cell Niche-Like Extracellular Matrix, Produced by Immature Astrocytes
Source: Front Cell Neurosci. 2020 May 21;14:102. doi: 10.3389/fncel.2020.00102 (PMC7253582; doi:10.3389/fncel.2020.00102)
Supplement: Supplementary file 4 [file Table_4.DOCX]

Supplementary Material

# Supplementary Table 4. Buffers and solutions, primers and PCR program for RT-PCR.

| **Buffer/Solution** | | **Source/Composition** | |
| --- | --- | --- | --- |
| Agarose gel (1 %) | | TAE buffer (1x)  1 % (w/v) agarose  ethidium bromide (1:10x10^6^) | |
| Loading buffer (6x) | | 200 µL 50x TAE  450 µL 0.2 M EDTA  3.5 mL glycerol  add aqua dest to 10 mL  add traces of bromophenol blue | |
| PCR buffer (10x) | | Sigma-Aldrich | |
| TAE buffer (50x) | | 242 g Tris base  57.1 mL acetic acid  100 mL 0.5 M EDTA (pH 8.0)  add aqua dest to 1 L | |
|  | | | |
| **Component for one PCR reaction** | | **Volume [µL]** | |
| cDNA | | 1.0 | |
| dNTP mix (10 mM each) | | 0.5 | |
| 10x Taq buffer | | 2.5 | |
| primer forward (10 pmol/µL) | | 1.0 | |
| primer reverse (10 pmol/µL) | | 1.0 | |
| Taq polymerase (5U/µL) | | 0.25 | |
| PCR water | | 18.75 | |
|  | | | |
| **Primer (from Sigma-Aldrich)** | **Sequence** | | **Amplicon (bp)** |
| β Actin for | TATGCCAACACAGTGCTGTCTGGTGG | | 247 |
| β Actin rev | AGAAGCACTTGCGGTGCACGATGG | |  |
| Tnc for | CTGCCAGGCATCTTTCTAGC | | 435 |
| Tnc rev | TTCTGCAGGTTGGAGGCAAC | |  |
|  | | | |
| **Time [min]** | **Temperature [°C]** | | **Number of cycles** |
| 2:40 initial denaturation | 94 | | 1 |
| 0:30 denaturation | 94 | | 25 |
| 0:35 annealing | 60 | |  |
| 1:00 elongation | 72 | |  |
| 5:00 final elongation | 72 | | 1 |
