# Supplementary figures and images for: Laser Lesion in the Mouse Visual Cortex Induces a Stem Cell Niche-Like Extracellular Matrix, Produced by Immature Astrocytes
Source: Front Cell Neurosci. 2020 May 21;14:102. doi: 10.3389/fncel.2020.00102 (PMC7253582; doi:10.3389/fncel.2020.00102)

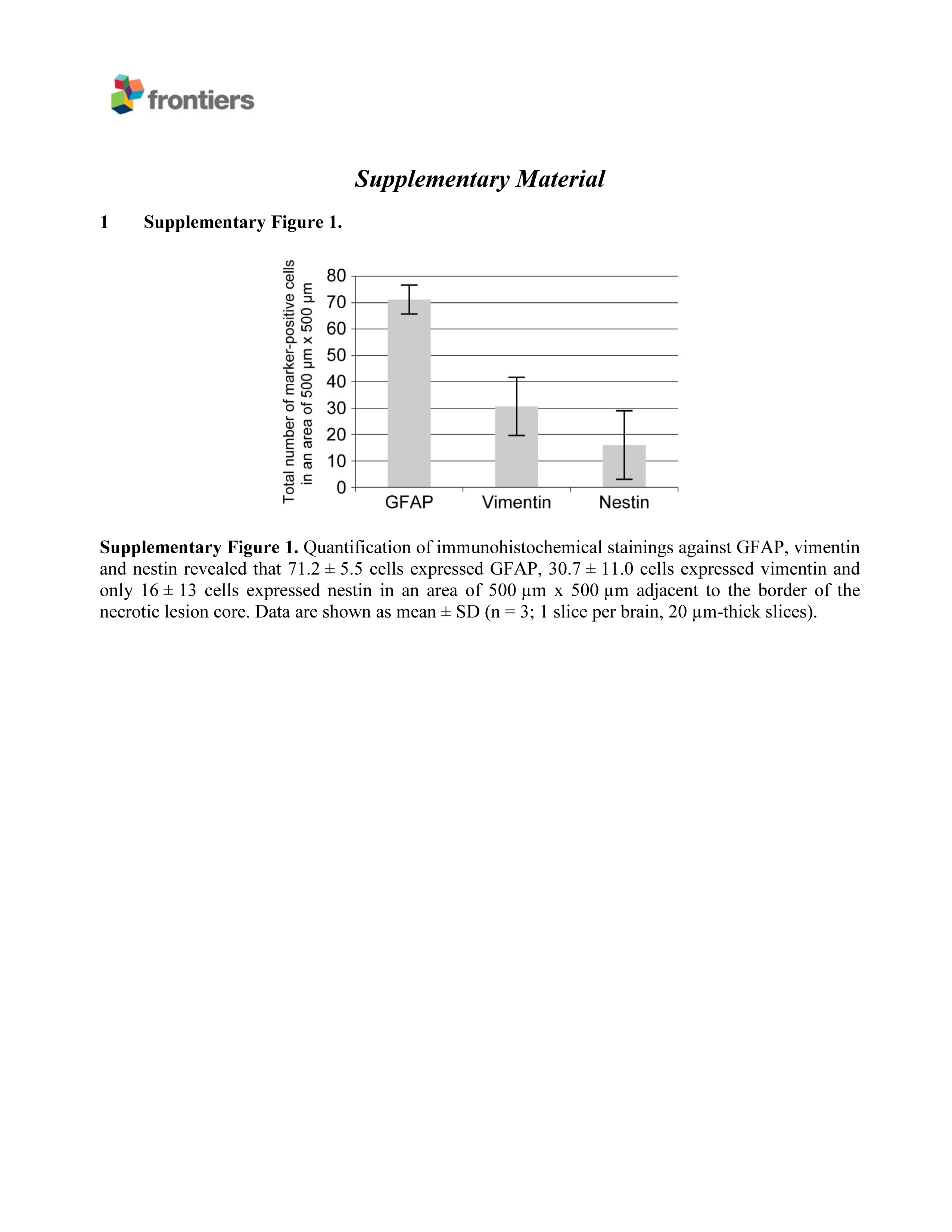

Supplement: Supplementary file 5 [file Image_1.JPEG]

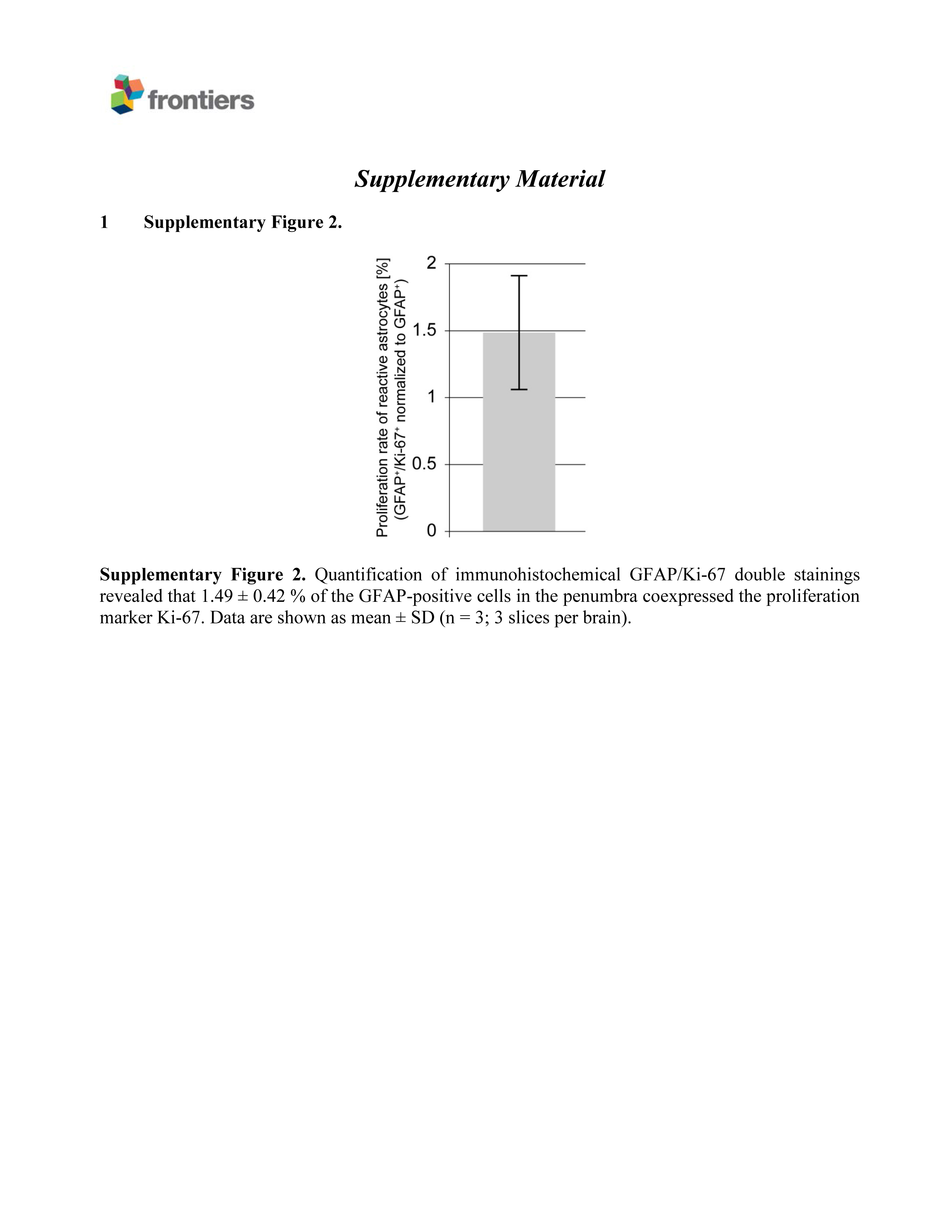

Supplement: Supplementary file 6 [file Image_2.JPEG]

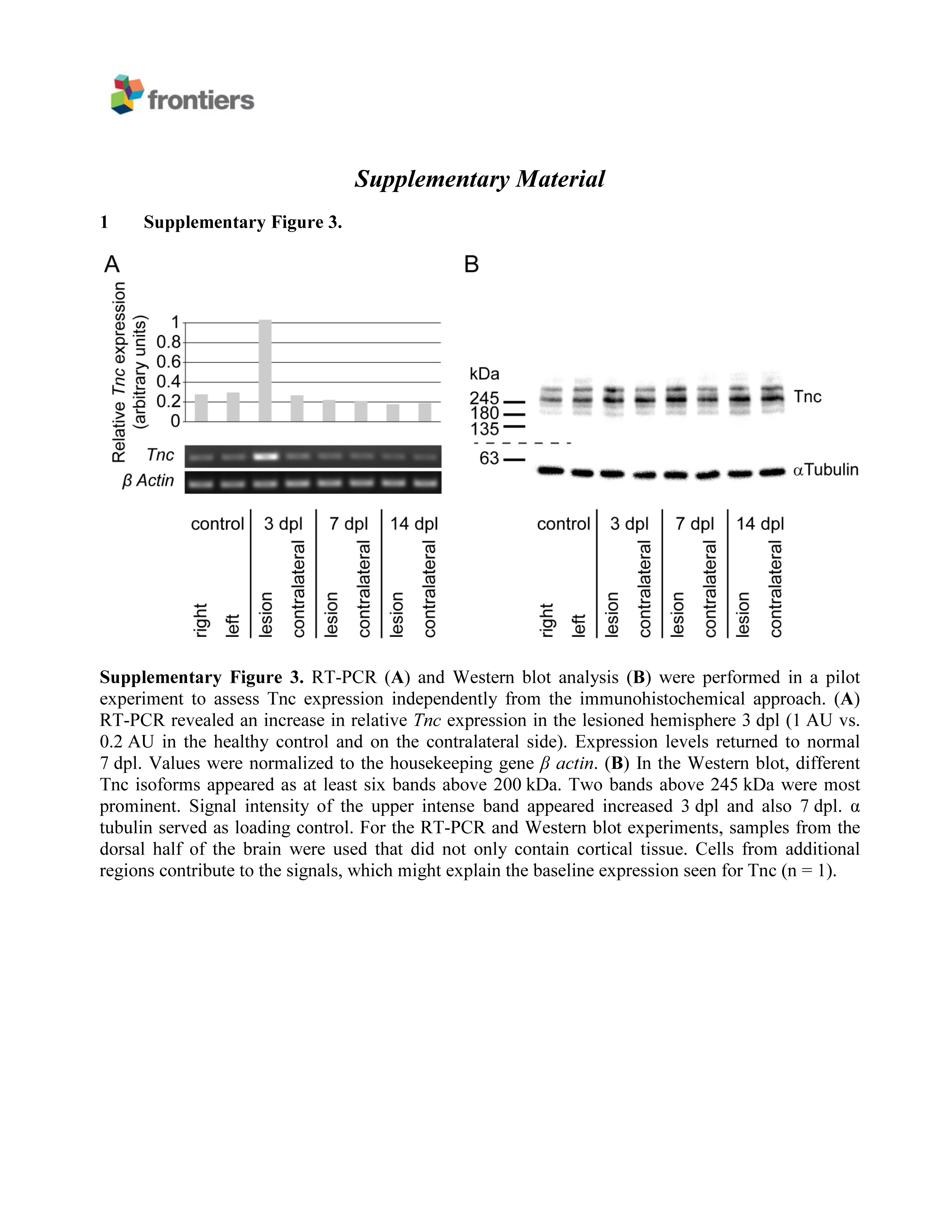

Supplement: Supplementary file 7 [file Image_3.JPEG]
